# Tracheobronchitis in patients with diffuse wall thickening: Three case reports

**DOI:** 10.1002/ccr3.5963

**Published:** 2022-06-09

**Authors:** Hirokazu Taniguchi, Yasuaki Masaki, Takeshi Tsuda, Hitoshi Abo, Atsushi Muto, Mami Shimizu, Naoki Takata, Akio Uchiyama, Akane Aikawa, Shin Ishizawa

**Affiliations:** ^1^ Department of Respiratory Medicine Toyama Prefectural Central Hospital Toyama Japan; ^2^ Department of Diagnostic Radiology Toyama Prefectural Central Hospital Toyama Japan; ^3^ Department of Pathology Toyama Prefectural Central Hospital Toyama Japan

**Keywords:** airway wall thickness, eosinophil, immunoglobulin G4, plasmacyte, tracheobronchitis

## Abstract

We herein report the cases of three patients with chest symptoms or fever and diffuse wall thickening of the trachea and main bronchi on chest CT. They were diagnosed with various causes of inflammations of the trachea and main bronchi using bronchial or tracheal biopsy specimens and flexible bronchoscopy.

## Graphical abstract

More attention should be paid to the wall of the trachea and main bronchi during the interpretation of chest CT in patients with chest symptoms and/or an inflammatory response.

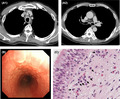

## 1 INTRODUCTION

Chest tightness, cough, and fever are common symptoms of many diseases. Many patients with these chief complaints are examined in medical facilities. Although bronchitis, bronchial asthma, cough‐variant asthma, or reflux esophagitis is often diagnosed, a lack of diagnosis is common. We suggest that these patients often have inflammation of the trachea and main bronchi.

Chest computed tomography (CT) is very useful in detecting chest diseases in patients presenting with chest symptoms or fever. Central airway abnormalities such as wall thickening are also easy to detect by chest CT.^1^, ^2^ Characteristic central airway wall thickening is typically observed in central airway amyloidosis, relapsing polychondritis, granulomatosis with polyangiitis, and adenoid cystic carcinoma, among other diseases.^1^, ^2^

Here, we present three patients with cough, chest tightness or fever exhibiting inflammation of the trachea, and main bronchi with diffuse wall thickening and without abnormalities in other organs. The patients were diagnosed with the aid of chest CT and bronchoscopy.

## 2 CASE PRESENTATIONS

### 2.1 Case 1

**FIGURE 1:**
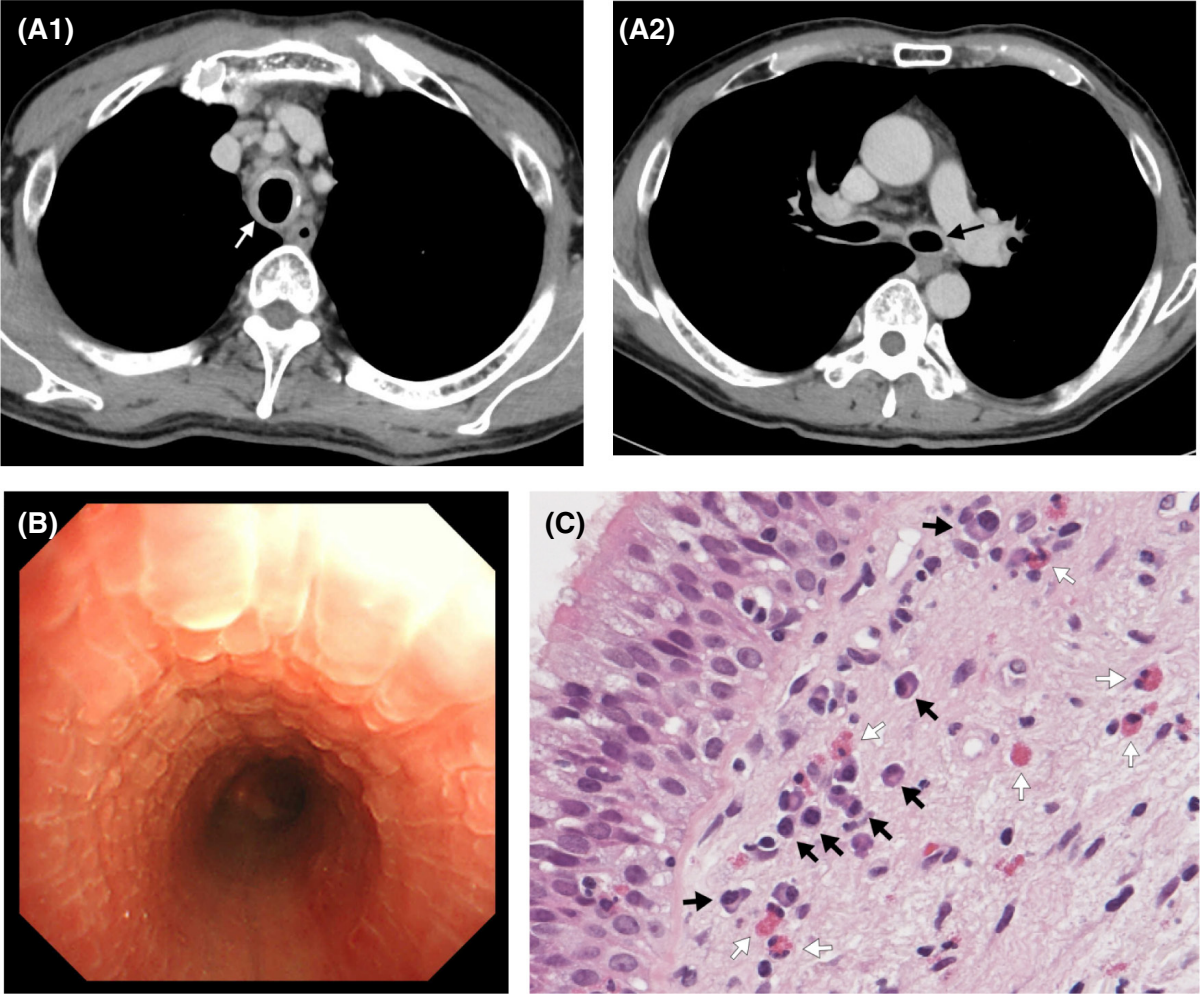
CASE 1: (A) Chest‐enhanced computed tomography revealed thickening of the tracheal wall (A1) and the main bronchi (A2) (arrow). (B) Flexible bronchoscopy showed an imbricate, edematous tracheal wall. (C) Bronchial biopsy specimens indicated bronchitis with moderate eosinophilic (white arrow) and mild plasmacytic (black arrow) infiltration (hematoxylin and eosin stain)

A 58‐year‐old Japanese man with a previous history of hypopharyngeal cancer visited Toyama Prefectural Central Hospital due to dry cough and chest tightness aggravated over 4 months. The patient was a current smoker and worked as a cook. No problems were noted upon auscultation. Blood test results showed an eosinophil count of 53 cells/μl, a C‐reactive protein level of 10.09 mg/dl (normal range: 0–0.14), and an immunoglobulin G4 (IgG4) concentration of 131 mg/dl (normal range: 5–117) (Table 1). Chest CT revealed moderate diffuse wall thickening of the trachea and main bronchi (Figure 1A). Flexible bronchoscopy detected an imbricate, edematous tracheal, and bronchial wall (Figure 1B). Bronchial biopsy specimens indicated airway inflammation with moderate eosinophilic and mild plasmacytic infiltration (Figure 1C, Appendix S1). His other organs were systematically examined for abnormalities, including CT of the head and trunk and blood and urine tests, but no abnormal findings were found.

**TABLE 1:** Clinical data of Case 1

Sex	Male	
Age	58	
Smoking	Smoking 20 cigarettes a day from 20 years of age to the first visit	
Profession	Cook	
Allergy history	None	
Previous history	hypopharyngeal cancer	

Blood test		Normal range
Hematology		
White blood cells	8.8 × 10^3^/μl	
Neutrophils	82.6%	
Eosinophils	0.6%	
Basophils	0.1%	
Lymphocytes	10.4%	
Monocytes	6.3%	
Red blood cells	3.88 × 10^6^/μl	
Hemoglobin	11.6 g/dl	
Platelets	476 × 10^3^/μl	
Biochemistry		
Total protein	7.6 g/dl	6.7–8.3
Lactate dehydrogenase	152 U/L	110–210
Aspartate aminotransferase	14 U/L	12–31
Alanine aminotransferase	10 U/L	8–40
Creatin phosphokinase	60 U/L	65–275
Blood urea nitrogen	10 mg/dl	8–22
Creatinine	0.65 mg/dl	0.60–1.10
Serology		
C‐reactive protein	10.09 mg/dl	0–0.29
Myeloperoxidase‐antineutrophil cytoplasmic autoantibodies	<0.5 U/ml	0–3.5
Proteinase3‐antineutrophil cytoplasmic autoantibodies	0.6 U/ml	0–2.0
Antinuclear antibodies	40	negative
Immunoglobulin G4	131 mg/dl	5–117
Immunoglobulin E	20 IU/ml	0–170

Prednisolone 0.5 mg/kg/day was administered; the patient's symptoms improved promptly, and the airway wall thickness exhibited a gradual reduction on chest CT after 4 months of treatment. The prednisolone dose was gradually reduced and stopped after 1 year of treatment.

### 2.2 Case 2

**FIGURE 2:**
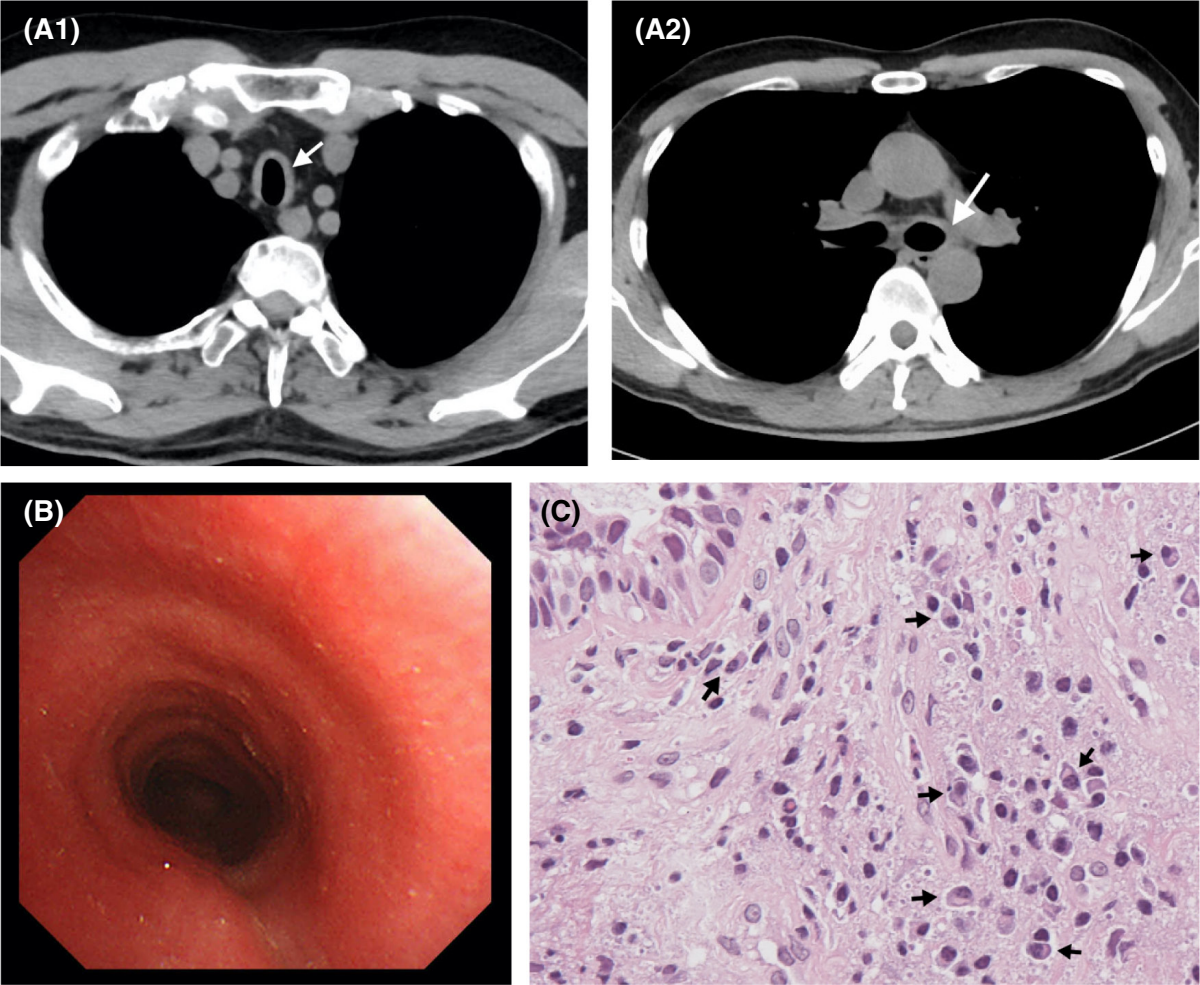
CASE 2: (A) Chest plain computed tomography revealed thickening of the tracheal wall (A1) and the main bronchi (A2) (arrow). (B) Flexible bronchoscopy showed an edematous tracheal wall. (C) Bronchial biopsy specimens indicated bronchitis with mild plasmacytic infiltration (arrow) (hematoxylin and eosin stain)

A 46‐year‐old Japanese man visited Toyama Prefectural Central Hospital due to dry cough that had persisted for 1 month. He was a current smoker. No problems were noted upon auscultation. Blood test results showed a C‐reactive protein level of 6.58 mg/dl and an IgG4 concentration of 191 mg/dl (Table 2). Diffuse wall thickening of the trachea and main bronchi was observed on chest CT (Figure 2A), and flexible bronchoscopy showed an edematous tracheal and bronchial wall (Figure 2B). Airway inflammation with mild plasmacytic infiltration was detected based on bronchial biopsy specimens (Figure 2C, Appendix S2). Most immunoglobulin G‐positive plasmacytes were positive for IgG4, and the IgG4/CD138 ratio was 0.56. His other organs were systematically examined for abnormalities, including CT of the head and trunk and blood and urine tests, but no abnormal findings were found.

**TABLE 2:** Clinical data of CASE 2

Sex	Male
Age	46
Smoking	Smoking 20 cigarettes a day from 20 years of age to the first visit
Profession	Sheet‐metal shop owner
Allergy history	None
Previous history	None

Blood Test		Normal range
Hematology		
White blood cells	6.8 × 10^3^/μl	
Neutrophils	68.8%	
Eosinophils	3.8%	
Basophils	0.4%	
Lymphocytes	21.6%	
Monocytes	5.4%	
Red blood cells	4.5 × 10^6^/μl	
Hemoglobin	12.7 g/dl	
Platelets	407 × 10^3^/μl	
Biochemistry		
Total protein	7.3 g/dl	6.6–8.1
Lactate dehydrogenase	126 U/L	124–222
Aspartate aminotransferase	26 U/L	13–42
Alanine aminotransferase	35 U/L	10–42
Creatin phosphokinase	57 U/L	59–248
Blood urea nitrogen	10 mg/dl	8–20
Creatinine	0.9 mg/dl	0.65–1.07
Serology		
C‐reactive protein	6.58 mg/dl	0–0.29
Myeloperoxidase‐antineutrophil cytoplasmic autoantibodies	<0.5 U/ml	0–3.5
Proteinase3‐antineutrophil cytoplasmic autoantibodies	0.6 U/ml	0–2.0
Antinuclear antibodies	Negative	Negative
Immunoglobulin G4	191 mg/dl	5–117
Immunoglobulin E	7 IU/ml	0–170

After 5 days, the patient developed severe cryptogenic hemoptysis. We treated him with methylprednisolone 1 g for 3 days, after which prednisolone 0.5 mg/kg/day was administered. The patient's symptoms disappeared promptly, and the airway wall thickness exhibited a gradual reduction on chest CT. The prednisolone dose was gradually reduced to 10 mg/day and was continued as a maintenance therapy to control his symptoms and inflammation.

He was challenging to diagnose as having IgG4‐related disease, even under the 2019 ACR / EULAR lgG4‐RD Classification Criteria.^3^

### 2.3 Case 3

**FIGURE 3:**
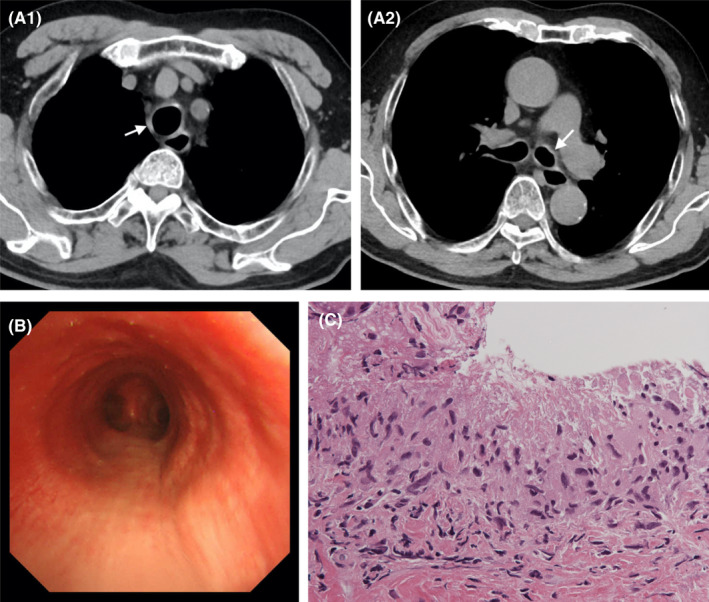
CASE 3: (A) Chest plain computed tomography revealed thickening of the tracheal wall (A1) and the main bronchi (A2) (arrow). (B) Flexible bronchoscopy showed an edematous tracheal wall. (C) Bronchial biopsy specimens indicated a palisading granuloma (hematoxylin and eosin stain)

A 76‐year‐old Japanese man with a history of bronchial asthma and chronic obstructive pulmonary disease for 2 years visited Toyama Prefectural Central Hospital due to a dry cough and approximately 38°C fever that lasted 2 weeks. No problems were noted upon auscultation. Blood test results showed an eosinophil count of 518 cells/μl, a C‐reactive protein level of 4.75 mg/dl, an IgG4 concentration of 51 mg/dl, and normal levels of myeloperoxidase and proteinase 3‐anti‐neutrophil cytoplasmic antibodies (Table 3). Chest CT showed diffuse wall thickening of the trachea and main bronchi (Figure 3A), and flexible bronchoscopy revealed an edematous tracheal and bronchial wall (Figure 3B). The bronchial biopsy specimens indicated tracheitis with palisading granuloma and multinucleated giant cells in the subepithelial area of the tracheal mucosa (Figure 3C, Appendix S3). His other organs were systematically examined for abnormalities, including CT of the head and trunk and blood and urine tests, but no abnormal findings were found.

**TABLE 3:** Clinical data of CASE 3

Sex	Male
Age	77
Smoking	Smoking 60 cigarettes a day from 20 to 36 years of age
Profession	Former office worker
Allergy history	Bronchial asthma
Previous history	Bronchial asthma and chronic obstructive pulmonary disease (75 years old)

Blood Test		Normal range
Hematology		
White blood cells	7.2 × 10^3^/μl	
Neutrophils	70.2%	
Eosinophils	7.2%	
Basophils	0.3%	
Lymphocytes	15.9%	
Monocytes	6.4%	
Red blood cells	4.46 × 10^6^/μl	
Hemoglobin	13.4 g/dl	
Platelets	334 × 10^3^/μl	
Biochemistry		
Total protein	6.8 g/dl	6.6–8.1
Lactate dehydrogenase	416 U/L	106–322
Aspartate aminotransferase	18 U/L	13–30
Alanine aminotransferase	18 U/L	10–42
Creatin phosphokinase	40 U/L	59–248
Blood urea nitrogen	17 mg/dl	8–20
Creatinine	1.15 mg/dl	0.65–1.07
Serology		
C‐reactive protein	4.75 mg/dl	0–0.29
Myeloperoxidase‐antineutrophil cytoplasmic autoantibodies	<0.5 U/ml	0–3.5
Proteinase3‐antineutrophil cytoplasmic autoantibodies	0.6 U/ml	0–2.0
Antinuclear antibodies	40	Negative
Immunoglobulin G4	51 mg/dl	5–117
Immunoglobulin E	13 IU/ml	0–170
Ferritin	187.2 ng/ml	50–200

We treated the patient with prednisolone 0.5 mg/kg/day. His symptoms disappeared promptly, and the airway wall thickness exhibited a gradual reduction on chest CT. The prednisolone dose was gradually reduced for 6 months and then stopped.

## 3 DISCUSSION

These case reports demonstrate that patients with cough, chest tightness, and fever may have inflammation of the trachea and main bronchi without abnormalities in other organs, which can constitute the pathogenesis of various clinical conditions. Previous reports have presented some cases of inflammation of the trachea and main bronchi with abnormalities in the lung and/or other organs, such as a case of IgG4‐related disease^4^, ^5^, ^6^ and a case of granulomatosis with polyangiitis.^7^, ^8^ However, there is no past report on inflammation localized to the trachea and main bronchi. We referred to this presentation as tracheobronchitis in the title because these three cases presented with lesions confined to the trachea and main bronchi.

In this series, CASE 1 showed eosinophilic and plasmacytic infiltration based on bronchial biopsy specimens, suggesting an allergic mechanism. He had a mild increase in plasma IgG4 and few IgG4‐positive cells in the airways. He was challenging to diagnose as having IgG4‐related disease, even under the 2019 ACR / EULAR lgG4‐RD Classification Criteria.^3^ For CASE 2, mild IgG4‐positive plasmacytic infiltration based on bronchial biopsy specimens was observed, indicating localized IgG4‐related disease under the 2019 ACR / EULAR lgG4‐RD Classification Criteria. CASE 3 exhibited palisading granuloma and multinucleated giant cells in the bronchial biopsy specimens, which led us to suspect a subtype of localized granulomatosis with polyangiitis. Although inflammation of the central airway was observed in all three cases, there is a distinct possibility that these three diagnoses had different underlying mechanisms.

Previously, it was not commonly considered that inflammation of the large airways would produce such diffuse wall thickening. However, in this report, we show that tracheobronchitis can cause various types of inflammation with wall thickening. It is suspected that tracheobronchitis is not a rare condition; however, it is likely less noticeable. It is challenging to diagnose because it is difficult to identify whether there is an abnormality only in that location. More attention should be paid to the wall of the trachea and main bronchi during the interpretation of chest CT findings in patients with chest symptoms or fever to fully understand their clinical condition. If the thickening of the airway wall is detected, we should investigate the cause of thickening with bronchial or tracheal biopsy using flexible bronchoscopy. The pathological findings in the biopsy tissue can reveal extensive airway conditions in these patients and guide appropriate treatment strategies.

It is also interesting why inflammation occurs locally in this area. The investigation of the causes of these pathologies is also interesting, smoking or inhalation of some substance may be involved. We hope that additional cases with similar findings are reported in the future to further our understanding of this clinical condition.

## AUTHOR CONTRIBUTIONS

HT drafted the manuscript and provided patient care. AU, AA, and SI performed pathological investigations. YM, TT, AM, MS, and NT performed bronchoscopy. HA created the computed tomography images.

## CONFLICT OF INTEREST

None declared.

## ETHICAL APPROVAL

This report was approved by the ethics committee at the Toyama Prefectural Central Hospital, and informed consent was obtained.

## CONSENT

Written informed consent was obtained from the patient to publish this report in accordance with the journal's patient consent policy.

## Supporting information


Appendix S1

Appendix S2

Appendix S3

## Data Availability

Data sharing not applicable to this article as no datasets were generated or analysed during the current study.
