# Supplementary material for: Tracheobronchitis in patients with diffuse wall thickening: Three case reports
Source: Clin Case Rep. 2022 Jun 9;10(6):e5963. doi: 10.1002/ccr3.5963 (PMC9178371; doi:10.1002/ccr3.5963)
Supplement: Supplementary file 1 — Appendix S1 [file CCR3-10-e5963-s003.docx]

Supplement 1: Histopathological findings of bronchial and tracheal biopsy specimens in CASE 1

1. Moderate eosinophilic infiltration and mild-to-moderate plasmacytic infiltration were revealed in bronchial biopsy specimens obtained during bronchoscopy at the bifurcation of the right upper lobe bronchus and intermediate bronchus. The eosinophils showed mild degranulation. Appropriate evaluation of the degree of immunoglobulin G4-positive cell infiltration was difficult due to strong background overstaining, but infiltration of a 10/1 high-power field or more was observed in the strongly infiltrated area. Oedema, amyloid deposits, basement membrane thickening, malignant cells and fungi were not found.

2. Mild eosinophilic infiltration and epithelial hyperplasia were revealed in tracheal biopsy specimens. Almost no plasma cells are found. The degree of eosinophil infiltration was weaker than that of biopsy tissue obtained during bronchoscopy at the bifurcation of the right upper lobe bronchus and intermediate bronchus.
