# Supplementary material for: Tracheobronchitis in patients with diffuse wall thickening: Three case reports
Source: Clin Case Rep. 2022 Jun 9;10(6):e5963. doi: 10.1002/ccr3.5963 (PMC9178371; doi:10.1002/ccr3.5963)
Supplement: Supplementary file 2 — Appendix S2 [file CCR3-10-e5963-s002.docx]

Supplement 2: Histopathological findings of bronchial and tracheal biopsy specimens in CASE 2

The pathological findings of the biopsy tissues from both the trachea and the bifurcation of the right upper lobe bronchus and intermediate bronchus revealed mild-to-moderate plasma cell infiltration under the bronchial mucosa, and some of these cells were collected. Appropriate evaluation of the degree of immunoglobulin G4-positive cell infiltration was difficult due to strong background overstaining, but IgG4-positive plasma cells were observed at a density of approximately 30/HPF. Most immunoglobulin G-positive plasmacytes were positive for IgG4, and the IgG4/CD138 ratio was 0.56. Increased fibre under the bronchial mucosa was found. Storiform fibrosis, thrombophlebitis, granulomas, basement membrane thickening, epithelial hyperplasia, oedema and malignant cells were not found.
