# Supplementary material for: Tracheobronchitis in patients with diffuse wall thickening: Three case reports
Source: Clin Case Rep. 2022 Jun 9;10(6):e5963. doi: 10.1002/ccr3.5963 (PMC9178371; doi:10.1002/ccr3.5963)
Supplement: Supplementary file 3 — Appendix S3 [file CCR3-10-e5963-s001.docx]

Supplement 3: Histopathological findings of bronchial and tracheal biopsy specimens in CASE 3

The pathological findings of the biopsy tissues from the trachea and the bifurcation of the right upper lobe bronchus and intermediate bronchus were fibrosis in the interstitium under the epithelium of the tracheal mucosa, granulomas with some histiocytes in a palisade array and multinucleated giant cells in some areas.

Eosinophilic necrotic tissue was found in the centre of the granuloma. Biopsy tissue with Elastica van Gieson staining showed no vascular structure around the granuloma and no evidence of vasculitis. Mild IgG-positive plasma cell infiltration was observed in the stroma under the tracheal mucosa, but IgG4-positive plasma cells were not observed. Basement membrane thickening, epithelial hyperplasia, oedema, fungi, acid-fast bacilli and malignant cells were not found.
